# Two New Species of *Ochotona* (Mammalia, Lagomorpha) From Xizang, China

**DOI:** 10.1002/ece3.71898

**Published:** 2025-08-06

**Authors:** Xuan Pan, Xuming Wang, Robert W. Murphy, Buqing Peng, Zongyun Zhang, Rui Liao, Shunde Chen, Shaoying Liu

**Affiliations:** ^1^ Sichuan Academy of Forestry Chengdu Sichuan China; ^2^ CAS Key Laboratory of Mountain Ecological Restoration and Bioresource Utilization & Ecological Restoration and Biodiversity Conservation Key Laboratory of Sichuan Province, Chengdu Institute of Biology Chinese Academy of Sciences Chengdu Sichuan China; ^3^ Department of Natural History Royal Ontario Museum Toronto Ontario Canada; ^4^ Sichuan Forestry and Grassland Survey and Planning Institute Chengdu Sichuan China; ^5^ Sichuan Normal University Chengdu Sichuan China

**Keywords:** *Conothoa*, molecular phylogeny, morphology, new species, *Ochotona*

## Abstract

During small mammal faunal surveys conducted in the Xizang Autonomous Region, China, in 2003, 2023, and 2024, we collected two previously unidentifiable taxa of pikas (*Ochotona*). Phylogenetic analyses based on both mitochondrial and nuclear gene sequences resolved these specimens as distinct lineages within the *O. macrotis* and *O. forresti* species groups, both in the subgenus *Conothoa*. Morphological comparisons further supported their uniqueness. Consequently, we described them here as two new species. One new species, *O. galunglaensis* sp. nov., collected from Motuo and Bomi counties, exhibits the following diagnostic characteristics: (1) diminutive body size (mean head‐body length < 150 mm); (2) reduced auricular dimensions (ear length < 18 mm); (3) summer pelage exhibits brown‐red on top of head, beneath eyes, and shoulders; neck bears a gray patch and ventral light brown‐gray; (4) without oval foramen; foramina incisivum narrow and palatal foramina merged; and (5) premaxillae on both sides of foramina incisivum have a tendency to close in some specimens. The other new species, *O. legbona* sp. nov., collected from Cuona county, is unique as follows: (1) comparable head‐body length to *O. macrotis* , but with much shorter ears (mean 23 mm vs. 31–35 mm in congeners); (2) characteristic black vibrissae lining the inner pinnae; (3) summer pelage exhibits brown‐red on head, shoulder, and neck; ventral brown‐yellow; and (4) without oval foramen; foramina incisivum narrow and palatal foramina merged. Our findings highlight the previously underestimated diversity within *Conothoa* and contribute to a more comprehensive understanding of pika diversity in the Himalayan region.

## Graphical abstract

During small mammals faunal surveys in Xizang conducted in 2003, 2023, and 2024, we collected two unidentifiable taxa of *Ochotona*. Phylogenetic analyses based on both mitochondrial and nuclear gene sequences resolve these specimens as distinct clusters within the *O. macrotis* and *O. forresti* species groups. Morphological comparisons support their uniqueness. Consequently, they constitute two new species.

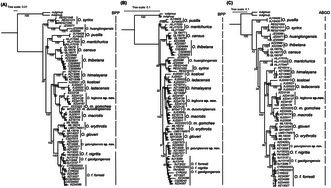

## 1 Introduction

Pikas (Lagomorpha: Ochotonidae) are small, mouse‐like lagomorphs with short limbs and rounded ears. Fossil records and ancient DNA evidence indicate that pikas were once widely distributed across Eurasia, Africa, and North America (Čermák 2004, 2010; Čermák and Rekovets 2010; Fostowicz‐Frelik and Frelik 2010; Čermák 2016; Rabiniak et al. 2023). During the Miocene, the family Ochotonidae comprised approximately 18 genera across the Holarctic, which declined to about five genera by the Pliocene (Wilson et al. 2016). Today, *Ochotona* is the only extant genus within the family.

Most extant pikas occur in China, particularly in the Hengduan Mountains, with a few inhabiting Central Asia, North America, and the Russian Far East (Lissovsky 2016; Smith et al. 2018). The genus currently includes at least 34 species, with ongoing discoveries such as *O. flatcalvariam* and *O. huanglongensis* (Liu et al. 2017), and species‐level recognition of taxa like *O. sikimaria* (Dahal et al. 2017). These species are classified into five subgenera: *Alienauroa*, *Conothoa*, *Lagotona*, *Ochotona*, and *Pika* (Liu et al. 2017; Tang et al. 2022), although the validity of monotypic *Lagotona* remains debated (Wang et al. 2020).

*Conothoa*, considered the earliest diverging subgenus within *Ochotona* (Wang et al. 2020; Tang et al. 2022), includes *O. erythrotis* , *O. forresti* , *O. iliensis, O. koslowi*, *O. ladacensis, O. macrotis, O. roylii, O. rufescens*, *O. gloveri* , and *O. rutila* (Table S1) (Lissovsky 2016; Smith et al. 2018). Species recognition within this subgenus remains contested, particularly regarding the taxonomic validity of *O. himalayana* and *O. chinensis* (Liu et al. 2017; Wang et al. 2020; Tang et al. 2022; Lissovsky et al. 2022); Lissovsky et al. (2022) synonymized both *O. himalayana* and *O. chinensis* into *O. roylii* and *O. macrotis* , respectively, but based on few mitochondrial and nuclear genes. To complicate matters, the distributional boundaries of *O. macrotis* and *O. roylii* remain unclear because some populations occur in remote border regions that challenge comprehensive sampling. Notwithstanding, Lissovsky et al. (2022) identified two main phylogenetic lineages within *Conothoa*: a western clade ( *O. macrotis* , *O. iliensis* , *O. roylii* , *O. rutila* , *O. ladacensis* , *O. koslowi* and *O. rufescens* ) and an eastern clade ( *O. forresti* and *O. erythrotis* ). They also reassigned the subspecies *O. forresti duoxionglaensis* to *O. macrotis* . Their taxonomic revisions require validation by deeper sampling and additional omics‐based research.

Species of *Conothoa* primarily occur in the Himalayan mountain characterized by extreme topographic and climatic variability. These mountains encompass three global biodiversity hotspots (Myers et al. 2000; Orme et al. 2005; Marchese 2015; Xing and Ree 2017). Numerous new species, subspecies, and speciation events have been described recently in this region (He et al. 2016; Li et al. 2017; Lissovsky et al. 2022; Wan et al. 2013; Ge et al. 2019; Liu et al. 2022; Chen et al. 2022), underscoring its importance as a center of evolutionary diversification. During zoological surveys conducted in Xizang, China, in 2003, 2023, and 2024, we identified two distinct taxa of *Conothoa*. Phylogenetic analyses based on seven genetic markers, combined with morphological comparisons, strongly suggest that these taxa represent new species. Herein, we focus on the identification and formal description of these two newly discovered species.

## 2 Materials and Methods

### 2.1 Ethics Statement

All sampling procedures strictly adhered to the American Society of Mammalogists (ASM) guidelines for animal care and use (Sikes and Animal Care and Use Committee of the American Society of Mammalogists, 2016) and were conducted in compliance with Chinese wildlife protection regulations (State Council Decree No. 13, 1992). Specimen collection protocols received formal approval from the Institutional Animal Care and Use Committee of the Sichuan Academy of Forestry (Ethics Approval Certificate No. SAFC‐IACUC/2018‐01). Voucher specimens were permanently archived at the Sichuan Academy of Forestry Zoological Collection (SAFZC), Chengdu, China.

### 2.2 Samples and Sequencing

**FIGURE 1:**
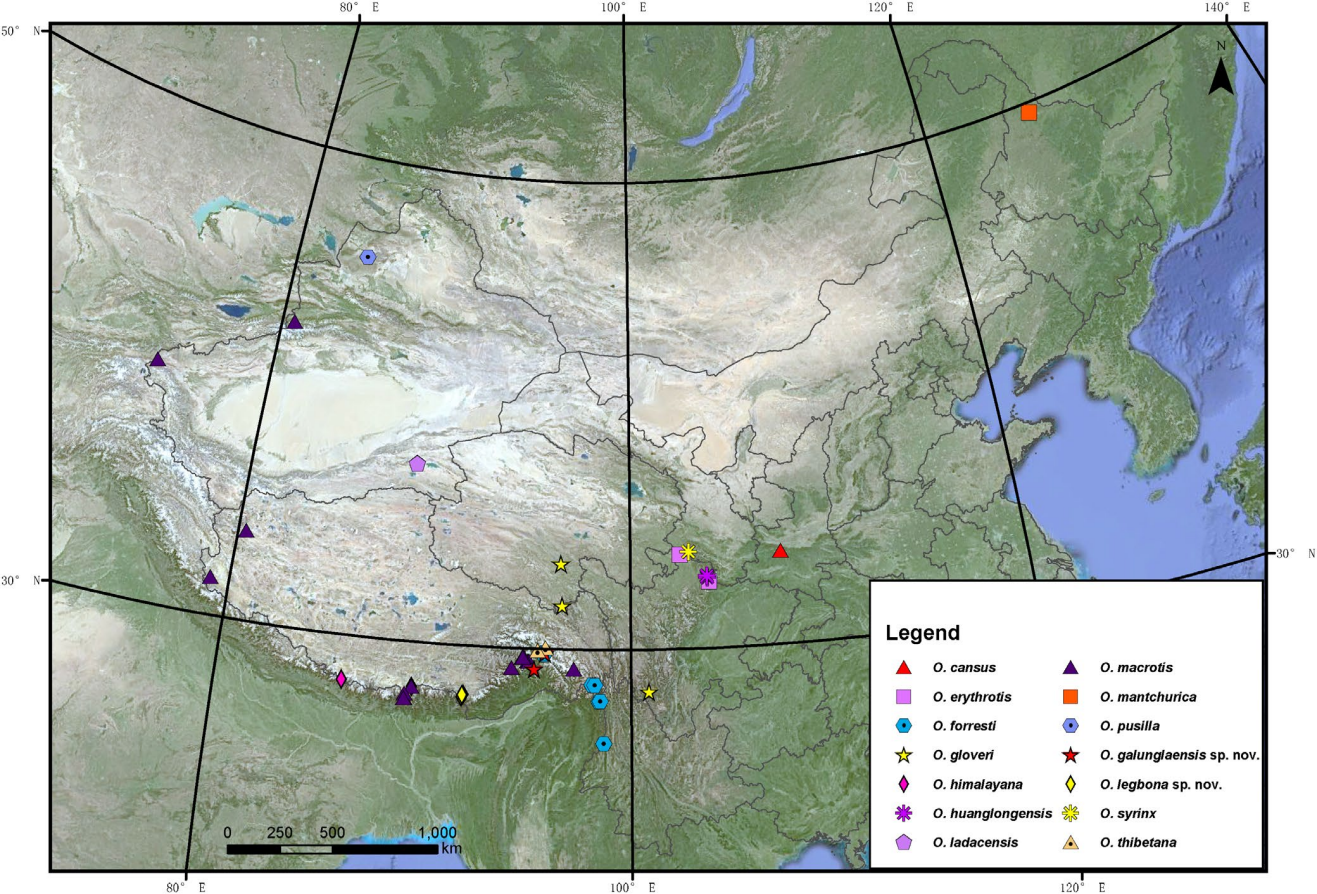
Map of sampling locations in the study.

Our molecular analyses incorporated tissues from 61 specimens representing 14 recognized species within the genus *Ochotona*, two unidentified *Ochotona* taxa, and two *Lepus sinensis* used as the outgroup. Specimen localities are shown in Figure 1, with complete collection details provided in Table S2.

All tissue samples were preserved in 95% ethanol and stored at −70°C until DNA extraction. Genomic DNA was isolated using the Animal Tissue Genomic DNA Rapid Extraction Kit (Chengdu Fuji Biotechnology Co. Ltd., Sichuan, China). We sequenced two mitochondrial genes (cytochrome b [*CYTB*, 1143 bp] and cytochrome c oxidase subunit I [*COI*, 917 bp]) and five nuclear loci (interleukin 1 receptor accessory protein‐like 1 [*ILRAPL1*, 1235 bp], oxidase assembly 1‐like protein [*OXA1L*, 1021 bp], titin [*TTN*, 1021 bp], recombination activating gene 1 [*RAG1*, 1021 bp], and recombination activating gene 2 [*RAG2*, 843 bp]). These markers were selected based on their established utility in resolving mammalian phylogenies (He et al. 2016; Koju et al. 2017; Lissovsky 2016).

PCR amplifications were performed in 25 μL reactions containing: 100 ng genomic DNA template, 5 pmol of each primer, 100 μM dNTPs, 2.5 μL 10× LA PCR Buffer, 1.25 U TaKaRa LA Taq (TaKaRa Biotechnology Co. Ltd., Dalian), and nuclease‐free water to volume. Thermal cycling parameters consisted of an initial denaturation at 95°C for 5 min; 34 cycles of 94°C for 30 s, 48°C–60°C for 50 s (annealing), and 72°C for 1.5 min (extension); followed by a final extension at 72°C for 10 min. Primer sequences and optimal annealing temperatures were adapted from published protocols (Teeling et al. 2000; Galewski et al. 2006; He et al. 2016; Cheng et al. 2017; see Table S3). Amplification success was verified by 1% agarose gel electrophoresis with ethidium bromide staining, using DL2000 markers (TaKaRa) for size determination. PCR products were purified using the MiniBEST DNA Fragment Purification Kit v3.0 (TaKaRa) and commercially sequenced (Sangon Biotech, Chengdu).

### 2.3 Phylogenetic Analyses

We established three datasets of the de novo data for phylogenetic analyses. The first dataset had concatenated *CYTB* and *COI*, the second concatenated all five nuclear genes, and the third concatenated all mitochondrial and nuclear genes. In addition, 63 published *CYTB* sequences of *Conothoa* were downloaded from GenBank to generate a fourth dataset containing 124 sequences that included all described species of *Conothoa*. Information for all sequences in this dataset is provided in Table S4.

All four datasets were aligned by MAFFT v7.526 with the auto‐alignment strategy (Katoh and Standley 2013). Phylogenetic analyses of the four datasets were performed using maximum likelihood (ML) analysis in RAxML‐NG v1.2.2‐master (https://github.com/amkozlov/raxml‐ng) and Bayesian inference (BI) in BEAST v1.7.4 (Drummond and Rambaut 2007). In RAxML, a tree was constructed using the GTRGAMMA substitution model, and the support value was assessed with a rapid bootstrap analysis of 1000 replicates followed by a thorough ML tree search. In MrBayes, two independent Markov Chain Monte Carlo (MCMC) analyses, each with four chains (three heated and one cold), were run for 5,000,000 generations, with sampling every 2000 generations. After graphical analysis of the evolution of likelihood scores, the first 25% of generations were discarded as burn‐in. The remaining trees were used to calculate the consensus tree. Convergence between runs was visualized in Tracer v1.7.2 (Rambaut et al. 2018). Tree visualization was conducted in FigTree v1.4 (https://github.com/rambaut/figtree) and iTOL (https://itol.embl.de/) (Letunic and Bork 2019).

### 2.4 Species Delimitation

To assess differentiation among species of *Conothoa* and populations within species, the Kimura‐2‐parameter (K2P) genetic distances for the *CYTB* dataset were calculated using MEGA v11.0 (Tamura et al. 2021), with standard error estimates obtained from 1000 bootstrap replicates. Automatic Barcode Gap Discovery (ABGD) and Bayesian Phylogenetics and Phylogeography (BPP) were used to test for species boundaries. ABGD was performed for the two mtDNA genes dataset and *CYTB* only using the K80 distance model and *X* values (0.25, 0.5, 0.75, and 1.0). The estimated transition/transversion bias was computed using MEGA, with other parameters set to default (*P*_min_ = 0.001, *P*_max_ = 0.1, steps = 10, bins = 20). The analysis was conducted online via a web page (https://bioinfo.mnhn.fr/abi/public/abgd) (Puillandre et al. 2012). BPP analyses (A10 and A11) were conducted with different θ (ancestral population size) and τ0 (divergence time) priors using datasets 2 (nuDNA) and 3 (mtDNA + nuDNA). Each run sampled 100,000 iterations (sampling every 50), discarding the first 20,000 as burn‐in. Lineages with posterior probabilities > 0.95 were considered independent species (Yang and Rannala 2010, Yang and Rannala 2014; Rannala and Yang 2013).

### 2.5 Morphology

The molecular results resolved two putative new species, one belonging to the *O. forresti* group and the other to the *O. macrotis* group. Therefore, morphological comparisons and analyses focused on these species. In total, 58 specimens representing three known species and both putative new species were selected from the collection at Sichuan Academy of Forestry (*O. macrtois*, *O. forresti* and *O. thibetana* ) (Table S5). Because the new species within the *O. forresti* group and *O. thibetana* occurred on both sides of the Yigong Zangbo River and had some similar morphological features (e.g., they did not have oval foramen on frontal bones), a specific morphological comparison between *O. galunglaensis* sp. nov. and *O. thibetana* was also carried out.

Abbreviations used in the morphological comparison followed Lissovsky et al. (2022) and Liu et al. (2017). External measurements, including head and body length (HB), ear length (EL), and hind foot length excluding the claws (HFL), were taken using a ruler to the nearest 0.1 mm. Sixteen cranial and dental characteristics were measured using a Vernier caliper to an accuracy of 0.02 mm, including the following skull measurements: greatest length (SGL), skull basal length (SBL), condylobasal length (CBL), zygomatic breadth (ZB), braincase width (WB), least interorbital width (IOW), nasal bone length (NBL), skull height (SH, from horizontal plane to the highest point when skull is placed horizontally with upper incisors and auditory bullae on ground), auditory bulla length (ABL), eye socket length (ESL), eye socket width (ESW), foramen incisivum length (FIL), length of maxillary tooth row (LMxT), mandibular tooth row length (LMbT), mandible length (ML, length of projected mandible on a flat surface including lower incisor), and lower mandible height (HLB). Principal component analyses (PCA) were then performed in R (v4.3.1) (http://www.r‐project.org/) to analyze skull features, with data visualization conducted using the ggplot2 package (Wickham 2011).

## 3 Results

### 3.1 Sequence Characteristics

After alignment, a total of 2801 bp mitochondrial DNA (*CYTB* [1259 bp] and *COI* [1542 bp]) and 4467 bp of nuclear genes (*IL1RAP1* [916 bp], *OXAIL* [961 bp], *RAG1* [1068 bp], *RAG2* [890 bp], and *TTN* [828 bp]) were obtained from 61 samples.

### 3.2 Phylogenomic Analyses

**FIGURE 2:**
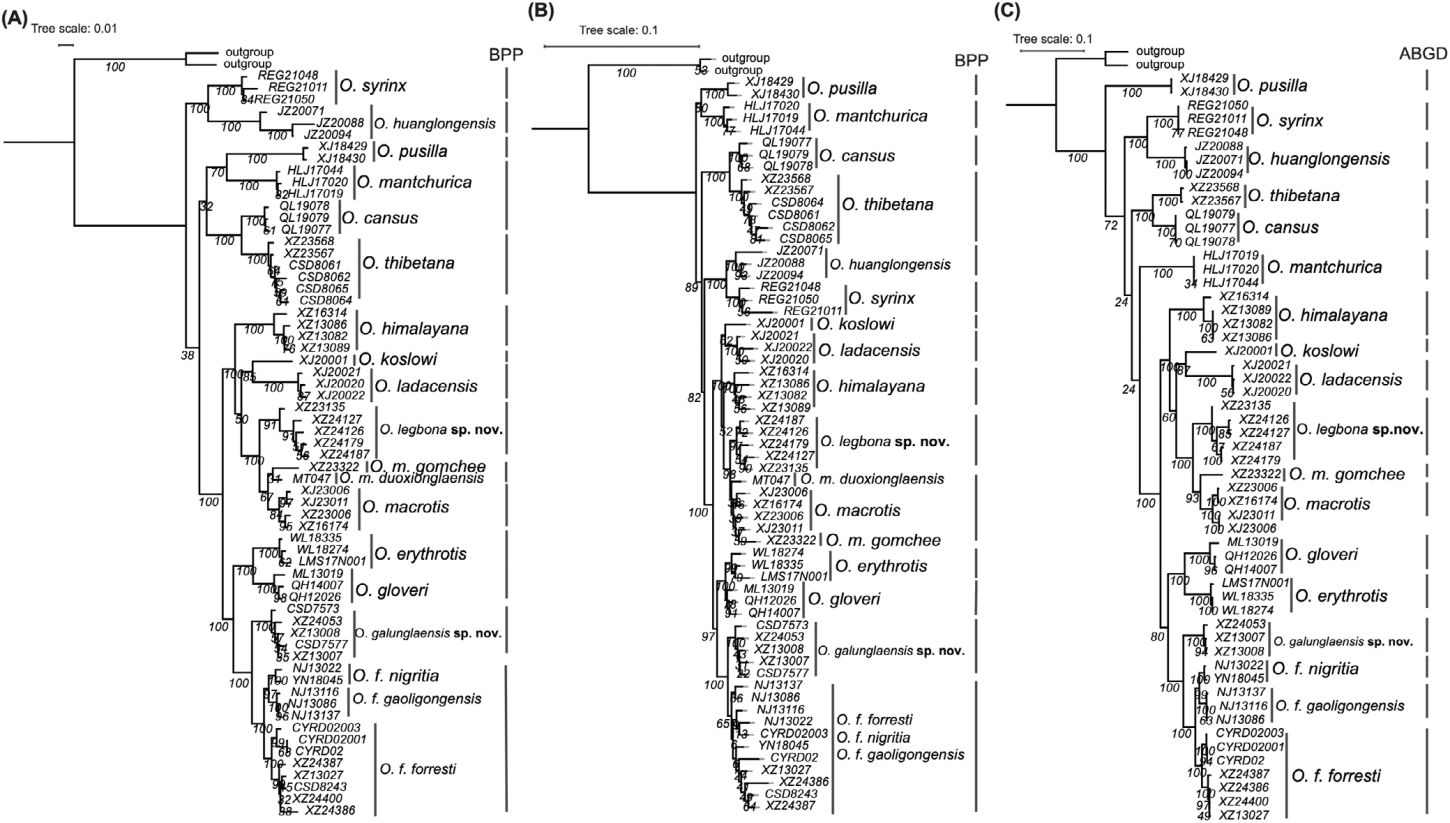
ML phylogenetic trees based on three datasets (A: MtDNA+nuDNA; B: NuDNA; C: MtDNA) (numbers at branches represent bootstrap values) and corresponding species delimitation results.

All trees consistently resolved subgenus *Conothoa* as sister to other *Ochotona* subgenera (*Pika*, *Lagotona*, *Ochotona*, and *Alienauroa*), while having seven major clades (Figure 2; Figure S1–S4). These lineages corresponded to: *O. gloveri* , *O. erythrotis* , the *O. forresti* group, *O. himalayana* , *O. koslowi* , *O. ladacensis* , and the *O. macrotis* group. Notably, concatenated mtDNA and combined mitochondrial‐nuclear datasets identified finer phylogenetic structure within the *forresti* and *macrotis* groups. The *forresti* group exhibited a basal divergence of *O. galunglaensis* sp. nov., followed by three subclades containing *O. f. forresti*, *O. f. gaoligongensis*, and *O. f. nigritia*. Similarly, the *macrotis* group showed a basal split of *O. legbona* sp. nov., with subsequent differentiation of *O. m. macrotis* and *O. m. gomchee* lineages. In contrast, nuclear gene concatenations yielded a simplified topology, resolving two primary clades within both the *forresti* and *macrotis* groups: one comprising *O. galunglaensis* sp. nov. and the other containing coalescent populations. CYTB tree displayed minor topological discordance for specific taxa (e.g., *O. himalayana* and the *forresti* group), as illustrated in Figure S4.

### 3.3 Species Delimitation

The mean intraspecific K2P distances within *Conothoa* (based on *CYTB*) was 12.2%, while ranging from 3.31% to 16.12% (Table 1). The minimum divergence (3.31%) occurred between *O. roylii* and *O. himalayana* , followed by 8.9% ( *O. erythrotis* vs. *O. gloveri* ), while *O. erythrotis* and *O. iliensis* had the maximum divergence (16.12%). Candidate new species exhibited intermediate differentiation from their sister taxa: *O. forresti* vs. *O. galunglaensis* sp. nov. (7.17%) and *O. macrotis* vs. *O. legbona* sp. nov. (7.05%) (Table 1).

**TABLE 1:** K2P distances between species of subgenus *Conothoa* based on *CYTB*.

	*O. erythrotis*	*O. gloveri*	*O. galunglaensis* sp. nov.	*O. forresti*	*O. rutila*	*O. koslowi*	*O. ladacensis*	*O. roylii*	*O. himalayana*	*O. iliensis*	*O. legbona* sp. nov.
*O. erythrotis*											
*O. gloveri*	8.9%										
*O. galunglaensis* sp. nov.	12.6%	12.1%									
*O. forresti*	11.5%	11.4%	7.2%								
*O. rutila*	13.9%	14.6%	13.3%	13.2%							
*O. koslowi*	14.3%	13.5%	11.9%	12.5%	10.7%						
*O. ladacensis*	15.0%	15.7%	14.9%	14.3%	13.8%	12.9%					
*O. roylii*	12.9%	13.3%	12.8%	12.1%	12.5%	12.4%	12.8%				
*O. himalayana*	13.1%	13.1%	12.7%	12.0%	12.5%	12.4%	13.3%	3.3%			
*O. iliensis*	16.1%	12.6%	11.4%	11.8%	13.0%	11.2%	14.9%	12.3%	11.3%		
*O. legbona* sp. nov.	12.1%	13.0%	12.2%	12.4%	12.3%	11.4%	13.9%	11.9%	11.7%	10.3%	
*O. macrotis*	11.8%	12.9%	11.6%	12.0%	11.2%	10.1%	12.9%	10.8%	10.8%	10.5%	7.1%

The BPP results for both datasets supported nine putative species (Figure 2), including *O. macrotis* , *O. roylii, O. gloveri*, *O. erythrotis* , *O. forresti*, *O. koslowi* , *O. ladacensis, O. galunglaensis* sp. nov., and *O. legbona* sp. no. The ABGD analysis identified 13 species, with the taxa within the *O. macrotis* group and the *O. forresti* group being further classified.

### 3.4 Morphology

**FIGURE 3:**
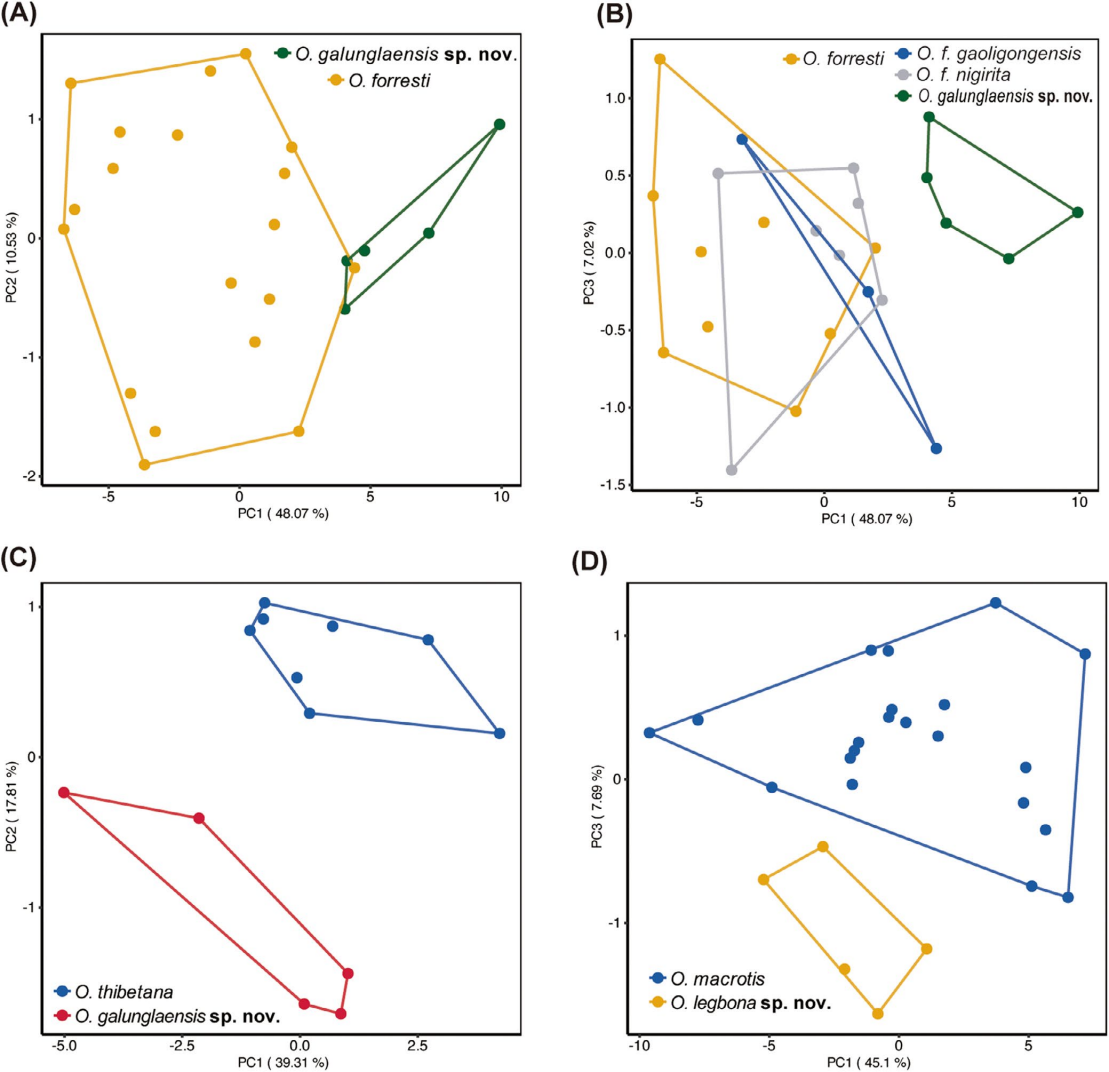
PCA plots of morphometric analysis based on 16 craniodental measurements. (A) *O. forresti* and *O. galunglaensis* sp. nov. (B) *O. forresti group*. (C) *O. galunglaensis* sp. nov. and *O. thibetana* . (D) *O. macrotis* and *O. legbona* sp. nov.

To assess morphological variation among the putative new species and their closely related species, a PCA analysis was performed using 16 cranial measurements (SGL, SBL, CBL, ZB, WB, IOW, NBL, SH, ABL, ESL, ESW, FIL, LMxT, LMbT, ML and HLB). The PCA clearly separated *O. forresti* from the putative new species (Figure 3A; Figure S5). Within *O. forresti* , *O. f. forresti*, *O. f. gaoligongensis*, and *O. f. nigirita* clustered together (Figure 3B). The first component (PC1) accounting for 48.07% of the total variance primarily reflected variation in SGL, CBL, and SBL. The second component (PC2) (10.53%) associated with SH, WB, ABL, and SBL, while the third component (PC3) (7.02%) was influenced mainly by ML and SGL. Similarly, the PCA distinguished putative *O. galunglaensis* sp. nov. from *O. thibetana* (Figure 3C; Figure S5). PC1 was driven by the difference in CBL, SBL, and SGL (39.31% of variation). PC2 (17.81%) reflected variation in NBL and ABL, while PC3 (8.82%) was shaped by HLB and ZB.

*O. macrotis* is clearly separated from putative *O. legbona* sp. nov. (Figure 3D; Figure S5). PC1 explained 45.1% of variation and was mainly influenced by SGL, SBL, CBL, and ML. PC2 (8.74%) was associated with ML, SH, ESL, FIL, and HLB, while PC3 (7.69%) reflected variation in ML, ESL, SH, and SBL.

## 4 Taxonomic Account

***Ochotona galunglaensis* Pan, Wang et Liu, sp. nov**. https://zoobank.org/NomenclaturalActs/58B0EBAA‐DB25‐4E8F‐A991‐DE3F00360482.

***Holotype***.—Adult male, summer pelage, field number CSD7526 (Museum number SCNU04481), was collected from Mêdog (Motuo) county, Xizang (Tibet), by Zhang Zongyun on June 3, 2024. The specimen was prepared as a skin with cleaned skull, deposited in Sichuan Academy of Forestry.

***Type locality***.—Motuo county, eastern Yarlung Zangbo River, 95.243425° E, 29.137985° N, 3000 m a.s.l.

***Measurements of Holotype**.—*Weight, 92.3 g; HBL, 161 mm; HFL, 27 mm; EL, 18 mm; SGL, 37.69 mm; SBL, 31.36 mm; CBL, 35.08 mm; ZB,18.36 mm; IOW, 4.25 mm; MB, 15.09 mm; SH, 13.23 mm; ABL, 9.65 mm; NBL, 11.13 mm; ESL, 86 mm; ESW, 7.75 mm; FICL, 9.73 mm; LMxT, 6.53 mm; LMbT, 6.54 mm; ML, 23.66 mm; MH, 14.28 mm.

***Paratypes**.—*Eight specimens (2 ♂♂, 6 ♀♀), skins with skulls, and male specimens with glans penis. Three specimens (CSD7524 (SCNU04479), ♂; CSD7575 (SCNU04530), ♀; CSD7598 (SCNU04553), ♂) from type locality, collected by Zhang Zongyun. Five specimens (XZ13007 (SAF13469), ♀; XZ13008 (SAF13470), ♀; XZ13034 (SAF13496), ♀, subadult, XZ13035 (SAF13497), ♀; XZ13037 (SAF13499), ♀) from 90 km northeastern of type locality, Bomi county, Xizang, 29.8231°–29.8432° N; 95.7552°–95.7631° E, 2900–3150 m, collected by Rui Liao on 29–30 October 2013.

***Additional specimens**.—*Five specimens (3 ♂♂, 2 ♀♀). XZ24053 (SAF24054), ♂, with skull broken, collected from type locality by Liao Rui and Zhang Zongyun on 30 March 2024; CSD7525 (SCNU04480), ♀, subadult, with skull broken; CSD7573 (SCNU04528), ♀, with skull broken; CSD7620 (SCNU04575), ♂, subadult, with skulls broken; CSD7577 (SCNU04532), ♂, with skull broken, are from the type locality, collected by Zhang Zongyun, from type locality on 3–5 June 2024.

***Geographic distribution***.—Known from Mêdog (Motuo) and Bomi counties, two sites about 90 km distant from each other (Figure 1). This region was limited to the north of Mount Mishmi and north and south of the Kangrigabo Mountains.

***Etymology***.—The species epithet is derived from one of the type localities: Galungla Mountain.

**FIGURE 4:**
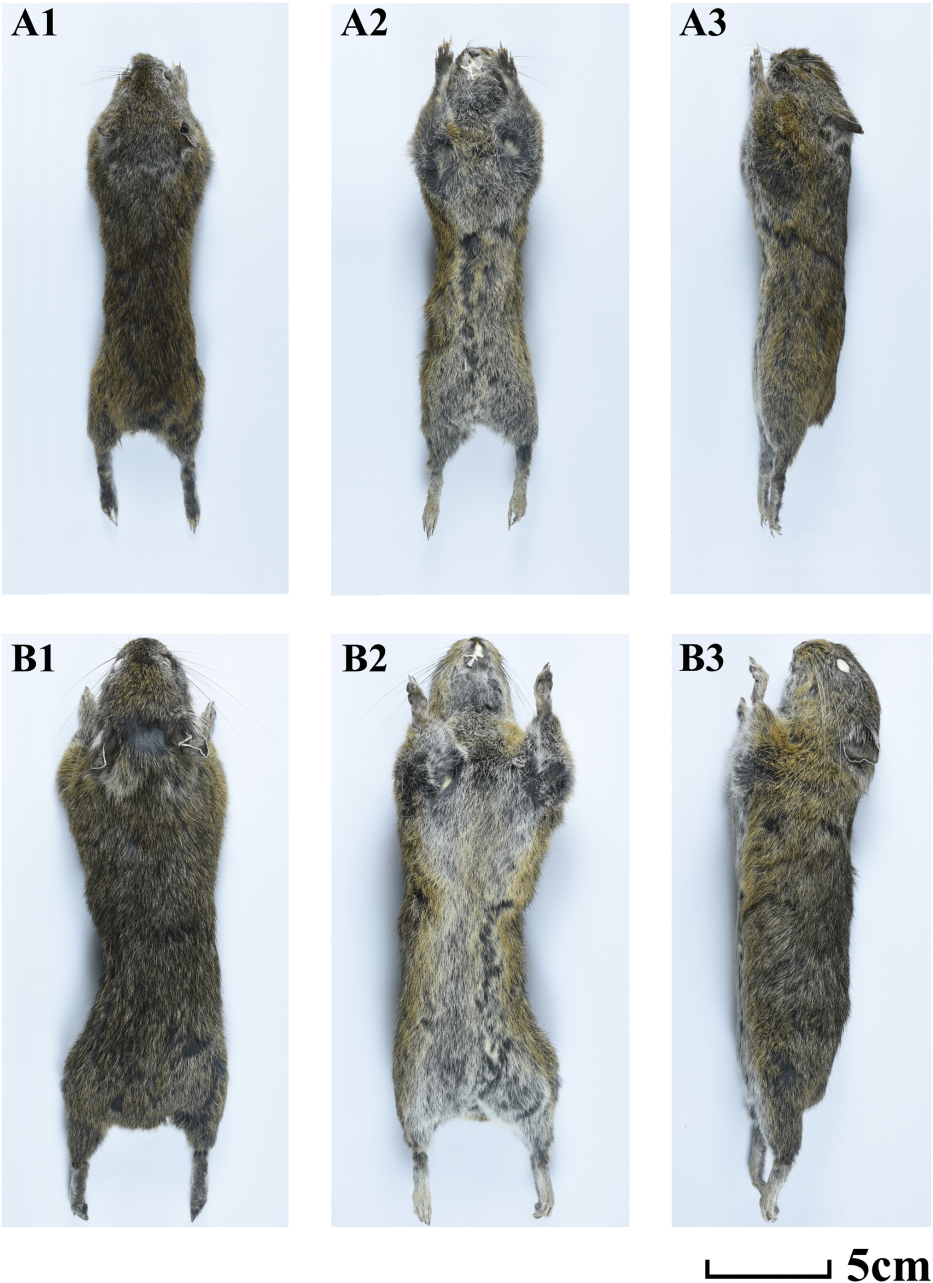
Dorsal, ventral, and lateral views of *O. galunglaensis* sp. nov. (A1–A3) and *O. legbona* sp. nov. (B1–B3).

**FIGURE 5:**
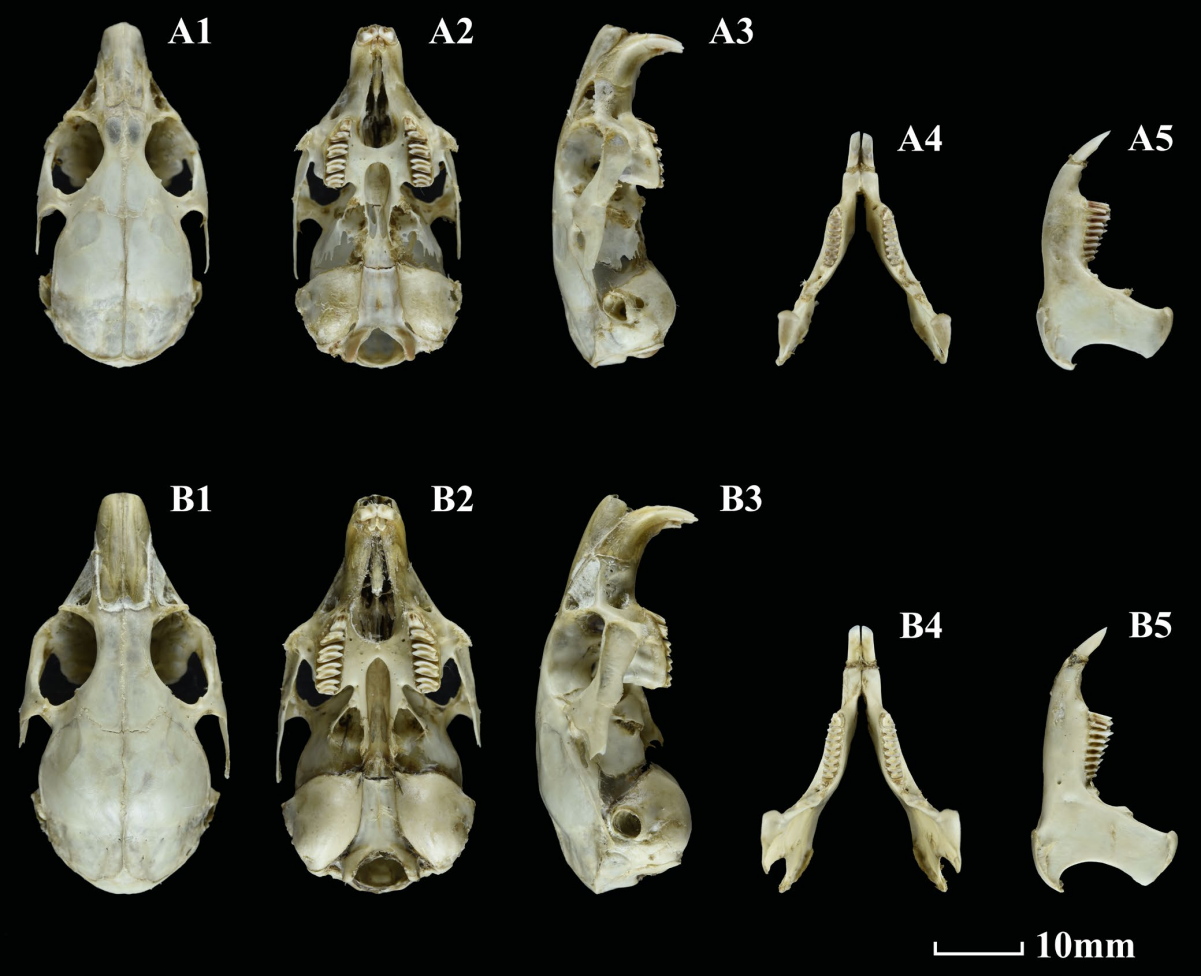
Dorsal, ventral, and lateral views of the skull, dorsal and lateral views of the mandible of *O. galunglaensis* sp. nov. (A1–A5) and *O. legbona* sp. nov. (B1–B5).

**FIGURE 6:**
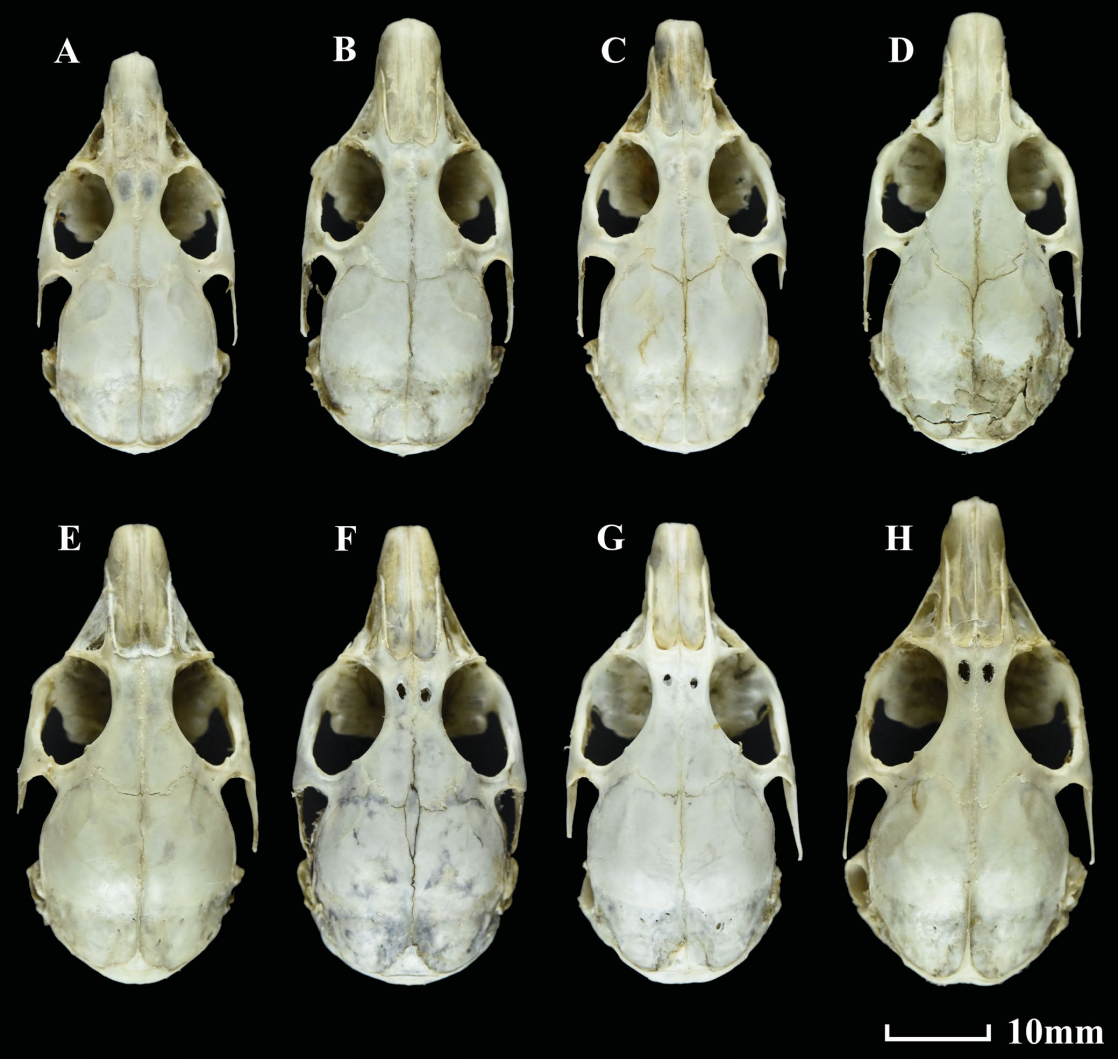
Dorsal views of the skull. (A) *O. galunglaensis* sp. nov.; (B) *O. forresti nigritia*; (C) *O. forresti gaoligongensis*; (D) *O. forresti* ; (E) *O. legbona* sp. nov.; (F) *O. macrotis gomchee* ; (G) *O. macrotis duoxionglaensis*; (H) *O. macrotis* .

***Diagnosis***.—A small pika, head and body length averaging less than 150 mm. Ear length very short, less than 18 mm. Summer pelage: top of head, underneath eye, shoulder brown‐red; neck with a gray patch; front of ear, entire back and side brown‐gray; before ear with brown tuft hairs; surrounding mouth with gray‐white hairs; chest brown; ventral light brown‐gray (Figure 4). Winter pelage: surrounding eye and cheek gray; neck with gray patch; before ear with brown tuft hairs; other parts of back brown; ventral brown‐yellow. Skull: no oval foramen (Figures 5 and 6); foramina incisivum narrow and merged with the palatal foramina. Some specimens with premaxillae on both sides of foramina incisivum tending to close.

***Description***.—General pelage of holotype (summer pelage) brown‐gray (Figure 4A). Top of head, underneath eye, and shoulder brown‐red; neck with gray patch; surrounding eyes gray‐white; check, front of ear, whole back, and side brown‐gray. Central back with many black pillar hairs. Ear very short, less than 18 mm, 17.5 mm on average. Inner side and back of ear black; the margin of ear white‐gray but very narrow. Front of ear with small brown‐red tuft. Gray‐white hairs surround mouth. Chin and throat gray, chest brown. Ventral hairs with black base and brown‐white tips. Transition between darker dorsal and lighter ventral pelage not abrupt. Back, front, and hind feet brown‐gray, and ventral of front and hind feet black. Claws brown‐white. Winter pelage: head, shoulder, whole back, and side with dull brown color. Neck with a gray patch. Margin of ear with very narrow gray‐white circle. Close to margin of ear with a narrow black circle, and surrounding external auditory canal light brown. Back of ear gray. Front of ear with a small brown‐red tuft. Chin and throat gray‐white; chest dull brown. Ventral hairs with black base and brown‐white tips. Back hairs of forelimb and hindlimb brown‐white. Ventral of forelimb and hindlimb black gray. Claws gray‐white. Digital pads large and brown‐white.

Mystacial vibrissae about 30 on each side, half white and half black or brown‐black. Shortest vibrissa about 5 mm, and longest about 40 mm.

Skull slightly slender (Figure 5), dorsal profile arc‐shaped, brain case elliptic. Nasals medium, sides of nasal parallel. Terminal of nasals circled and pointed, inserted in front of frontal. Posterior and anterior of frontal broad, while narrow in the middle. Inner side of posterior orbit with a very small projection. Outside of parietal circled, with mid‐anterior point protruding to frontals. Interparietal bone very small, trapezoid‐like. Anterior interparietal with a very large ridge. Zygomatic arches medium, posterior end pointed. Foramina incisivum narrow, merged with palatal foramina. Some specimens with premaxillae on both sides of foramina incisivum tend to close. Auditory bullae medium, circular. Mandible with broad and trapezoid‐like zygopophysis and circular‐curved processus angularis.

Dental same as in *O. legbona* sp. nov. but smaller. Dental formula 2.0.2.3/1.0.2.3 (Figure 5). First upper incisors white and with a deep longitudinal groove. Second upper incisors very small, located in the interior of the first upper incisors. First upper premolar very small, front surface with two shallow grooves, masticatory surface with a groove and humps. Second upper premolar larger than the first premolar, slightly smaller than the first and second molars, masticatory surface with a deep groove and 2 humps. First and second upper molars equal sized. Masticatory surface of the first and second molars with 2 deep grooves and transversal ridges. Third upper molar smaller than the first and second molars, also with 2 deep grooves and transversal ridges.

Lower incisors white. Labial side of first lower premolar with 2 longitudinal ridges, lingual side with a longitudinal ridge, and masticatory surface triangular with 2 short grooves and 2 humps. Appearance of second lower same as first and second molar, masticatory surface, constituting 2 triangles. Third lower molar much smaller, masticatory surface with a single triangle.

***Comparison***.—Compared with sympatric *O. forresti* , *O. galunglaensis* sp. nov. is much smaller, with the smallest head and body length of *O. forresti* being more than 170 mm, but the largest head and body length of *O. galunglaensis* sp. nov. being less than 160 mm. Pelage in *O. forresti* (winter pelage) has a brown forehead and a much duller back color (gray), but *O. galunglaensis* sp. nov. (winter pelage) brown. The ventral pelage of *O. forresti* is gray‐white, but *O. galunglaensis* sp. nov. has brown‐yellow ventral hairs.

Compared with *O. forresti gaoligongensis* and *O. forresti nigirita*, the summer pelage of *O. galunglaensis* sp. nov. is much duller, with only the head and shoulder being brown‐red. In *O. forresti gaoligongensis* and *O. forresti nigirita*, the summer pelage of the entire back is more red. The ears of *O. forresti gaoligongensis* and *O. forresti nigirita* are much longer than those of *O. galunglaensis* sp. nov.

Compared with sympatric *O. thibetana* (north of Yigong Zangbo River), *O. galunglaensis* sp. nov. has a much shorter ear length; the pelage (winter) of *O. galunglaensis* sp. nov. is much browner, and the winter pelage of *O. thibetana* is deep gray. The inner side of the ear of *O. thibetana* has long white hair, but in *O. galunglaensis* sp. nov., it has light brown hairs that are close to the margin black‐brown. The back of the forelimb and hindlimb of *O. thibetana* is white, but *O. galunglaensis* sp. nov. has brown‐gray. The skull of *O. thibetana* has a much broader nasal bone; *O. galunglaensis* sp. nov. has longer foramina incisivum.

***Habitat***.—The type locality is characterized by bamboo vegetation approximately 3 m tall, with a coverage of about 75%. The other locality consists of pristine coniferous forest dominated by spruce trees, which reach up to 20 m in height and have a canopy coverage of approximately 30%. Both localities feature thick humus layers and loose soil. The burrows of this species are typically around 20 mm in diameter and are usually found beneath decaying logs.

***Ochotona legbona* Pan, Wang et Liu, sp. nov**.

https://zoobank.org/NomenclaturalActs/D477DFAB‐A22D‐48B4‐8069‐22D0CFCC601A.

***Holotype**.—*Adult male, field number XZ24179 (Museum number SAF240179) (winter pelage) collected from Legbo Valley, Cuona county, Xizang, by Rui Liao and Zhang Zongyun on 10 April 2024. Specimen prepared as a skin with cleaned skull and deposited in Sichuan Academy of Forestry.

***Type locality***.—Legbo valley, Cuona (Cona) county, 27.89872° N; 91.80032° E, 2970 m a.s.l.

***Measurements of holotype**.—*Weight, 146 g; HBL, 185 mm; HFL, 33 mm; EL, 23 mm; SGL, 42.55 mm; SBL, 35.34 mm; CBL, 38.96 mm; ZB, 21.75 mm; IOW, 5.42 mm; MB, 17.96 mm; SH, 15.86 mm; ABL, 10.88 mm; NBL:14.01 mm; ESL, 14.01 mm; ESW, 8.86 mm; FICL, 11.38 mm; LMxT, 7.32 mm; LMbT, 7.28 mm; ML, 27.77 mm; MH,16.69 mm.

***Paratypes***.—4 specimens (4 ♂♂), skins with skulls (XZ23135 (SAF230515), ♂, summer pelage; XZ24126 (SAF240126), ♂, winter pelage; XZ24127 (SAF240127), ♂, winter pelage; XZ24187 (SAF240187), ♂, winter pelage) from near the type locality. Elevation 2970–4350 m a.s.l.

***Geographic distribution***.—Presently known only from Cuona (Cona) county (Figure 1).

***Etymology***.—The species epithet is derived from the Legbo Valley.

***Diagnosis***.—A pika belonging to the *O. macrotis* group. Head and body length similar to *O. macrotis* . Ear length averaging 23 mm, much shorter than in *O. macrotis* . Inside of ear with short black hairs. No oval foramen on frontal bones. Summer pelage: head, shoulder, neck brown‐red; chin and throat black‐gray; posterior of back black‐gray; ventral brown‐yellow. Winter pelage: top of head, cheek, shoulder, close to ventral part of body light brown; other parts of body brown‐gray. Foramina incisivum narrow and merged with the palatal foramina. In some specimens, premaxillae on both sides of foramina incisivum tend to close.

**FIGURE 7:**
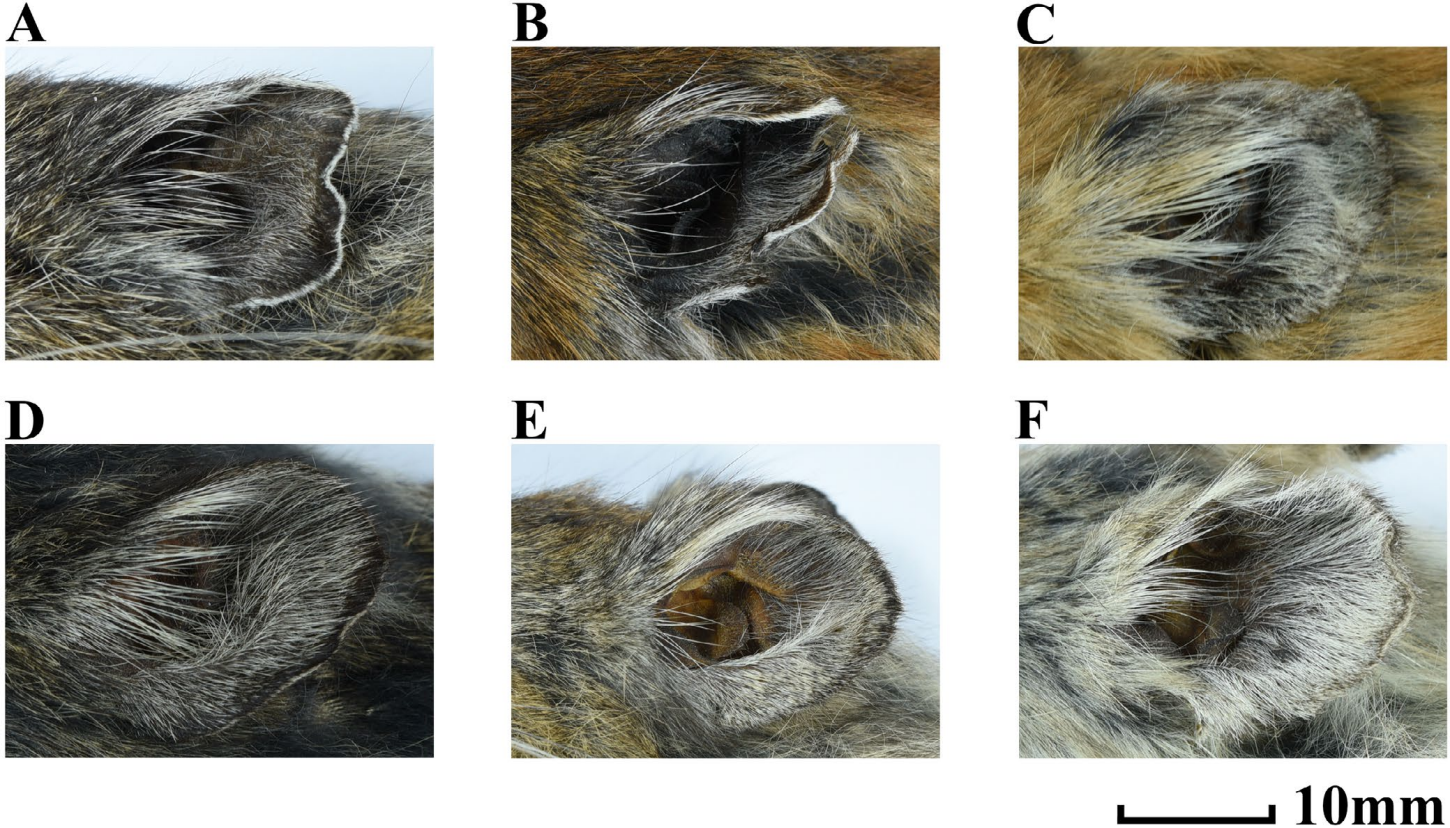
Auricle morphology of *O. legbona* sp. nov. and *O. macrotis* . (A) *O. legbona* sp. nov. in winter pelage; (B) *O. legbona* sp. nov. in summer pelage; (C) *O. macrotis* in summer pelage, shrubland habitat; (D) *O. macrotis* in summer pelage, coniferous forest; (E) *O. macrotis* in summer pelage, broadleaf forest; (F) *O. macrotis* in summer pelage, scree habitat.

***Description***.—Holotype general winter pelage brown‐gray (Figure 4B). Top of head, cheeks, shoulders, and sides near ventral brown. Gray around eyes. Ear short, averaging 23 mm. Hairs inside ear short and black. Margin of ear white. Back of ear with a slightly duller patch. Back brown‐gray, base of fur black, tip brown‐gray. Venter lighter than dorsum, with yellow‐white hairs. Chin and throat gray‐white with black base. Chest brown. Lips black. Transition between dorsal and ventral pelage not abrupt. Summer pelage: head, cheeks, and shoulders brown‐red, posterior back black‐gray, chin and throat gray‐white with black base. Chest light brown‐red color. Ventral hairs with black base and light brown tips. Ear same as winter pelage (Figure 7A,B).

Mystacial vibrissae mostly white, but some black, about 20 on each side. Shortest vibrissa about 10 mm, and longest about 60 mm. Back hairs of forelimb and hindlimb yellow‐white or gray‐white. Ventral black gray. Claws black‐gray. Digital pads large and gray.

Skull sturdy (Figure 5B), dorsal profile arc‐shaped, brain case elliptic. Nasals broad, sides parallel. Posterior margin of nasals cambered, contacting front of maxilla. Posterior and anterior frontal broad, narrow in middle. Outside of parietal cambered, with mid‐anterior point protruding to frontals, back of parietal uplifted. Interparietal very small and triangular. Anterior of interparietal bone had a ridge. Zygomatic arches sturdy, posterior end pointed. Foramina incisivum narrow and merged with palatal foramina. In some specimens, premaxillae on both sides of foramina incisivum tend to close. Auditory bullae large and triangular. Mandible with broad and high zygopophysis and circular‐curved processus angularis.

Dental formula 2.0.2.3/1.0.2.3 (Figure 5B). First upper incisors white with a longitudinal groove. Second upper incisors very small, located between first upper incisors. First upper premolar smaller, masticatory surface with groove and humps. Second upper premolar larger than first premolar and slightly smaller than first and second molar, masticatory surface with a deep groove and 2 humps. First and second upper molar equal sized. Masticatory surface of first and second molars with 2 deep grooves and transversal ridges.

Lower incisors medium and white. Labial side of first lower premolar with 3 longitudinal ridges, masticatory surface triangular. Second lower is the same as first and second molar, masticatory surface with 2 triangles. Third lower molar is much smaller, masticatory surface with a single triangle.

***Habitat***.—Terra typica vegetation consists of secondary coniferous forest dominated by spruce trees approximately 6 m in height, with a canopy coverage of about 40%. Understory vegetation includes bamboo shrubs around 1.5 m tall, covering approximately 70% of the area. The second site is characterized by bamboo shrubland, also about 1.5 m in height, with a coverage of around 50%. Both sites are located in valley shrublands featuring large rocks and sandy soil. Burrows are typically found beneath rocks or among bamboo clumps.

***Comparison***.—Compared with *O. macrotis* , the summer pelage is less variable. Specimens of *O. macrotis* in talus have light brown‐yellow anterior parts of the body and posterior parts of the body gray‐white mingled with brown‐yellow patches; specimens inhabiting forests have black‐colored hairs on the entire body; specimens occupying bushes have brown tops of the head and shoulders, with black patches mingled in the gray back. The inner side of the ear in all specimens has long, white hairs (Figure 7A–F).

Compared with *O. macrotis duoxionglaensis*, *O. macrotis duoxionglaensis* has much duller summer pelage, only forehead brown‐red. The entire head and neck of *O. legbona* sp. nov. are covered with light brown‐red hairs. The inner side of the ear of *O. macrotis duoxionglaensis* has short white hairs, but *O. legbona* sp. nov. has back hairs. There is no oval foramen in *O. legbona* sp. nov., but *O. macrotis duoxionglaensis* has a very small pair of oval foramina (Figure 6).

Compared with *O. macrotis gomchee* , *O. legbona* sp. nov. does not have an oval foramen on the frontal. The inside of the ear of *Ochotona legbona* sp. nov. has black short hairs, but *O. macrotis gomchee* has short gray‐white hairs. The pelage in *O. macrotis gomchee* (summer pelage) much duller, with only the forehead and cheek having brown hairs, but in *O. legbona* sp. nov., the entire head, including the neck light brown‐red. The venter of *O. macrotis gomchee* is gray‐white, but *O. legbona* sp. nov. is light brown.

## 5 Discussion

Our results have notable implications for the systematics and taxonomy of the genus *Ochotona*, particularly within the subgenus *Conothoa*. First, our results support the recognition of two previously undescribed species. Although specimens of these taxa had been collected and partially analyzed in earlier studies, they were not previously recognized as distinct. For example, Lissovsky et al. (2022) examined two specimens from Bomi and placed them within the *O. forresti* group based on molecular data, noting significant differentiation but relatively low maximum likelihood distances. Consequently, those specimens were not recognized as a distinct species. However, our expanded dataset and detailed morphological comparisons provide robust evidence for recognizing them as a distinct species, which we name *O. galunglaensis* sp. nov. Similarly, Dahal et al. (2020) identified eastern Himalayan pika populations as *O. macrotis* in a study focused on ecological and geographic patterns rather than taxonomy. Our integrative approach—combining broader molecular sampling with morphological data—demonstrates that this population represents a distinct evolutionary lineage, now described as *O. legbona* sp. nov. These discoveries underscore the underestimated diversity within *Conothoa* and highlight the need for broader geographic sampling and multigene data analyses to clarify species boundaries in regions of high biodiversity.

Second, we propose that the presence or absence of an oval foramen on the frontal bone is a valuable morphological character in pika taxonomy. While previously considered variable and taxonomically insignificant, we have consistently observed oval foramen in species such as *O. erythrotis* , *O. gloveri* , and *O. macrotis* across nearly two decades of specimen collection, despite some variation in size. Importantly, the absence of this feature in *O. legbona* sp. nov. provides a clear morphological distinction from other *O. macrotis* specimens, strengthening its taxonomic recognition as a distinct species when considered alongside molecular and other species delimitation evidence.

Third, our results suggest that the phylogenomic structure of *O. macrotis* requires further revision. This species is widely distributed across Central and South Asia, including China, Kazakhstan, India, Nepal, and Bhutan, and was once recognized to have three subspecies: *O. m. chinensis*, *O. m. macrotis*, and *O. m. gomchee* (Lissovsky et al. 2022). Recent genomic analyses have elevated *O. m. chinensis* to species status (Wang et al. 2020; Tang et al. 2022). Our species delimitation analysis using BPP also suggests the recognition of *O. m. gomchee* as a distinct species, although Lissovsky (2016) described it as a subspecies due to the cranial shape similarity to *O. macrotis* and moderate genetic divergence (cytb K2P distance of 8.2%). Furthermore, our field observations indicate that *O. m. gomchee* occurs sympatrically with *O. macrotis* without hybridization or intermediate forms, suggesting reproductive isolation. Although our current sample size is limited, these findings justify further genetic and ecological investigations to confirm its species status.

In summary, our integrative approach—combining multilocus molecular data with detailed morphological analyses—supports the recognition of 12 species within the subgenus *Conothoa*. This finding highlights the previously underestimated diversity of pikas in the Himalayan region and contributes to a more comprehensive understanding of their taxonomy. While the current multilocus methods remain useful for resolving species boundaries, more comprehensive phylogenomic analyses are essential to fully clarify the evolutionary relationships within *Ochotona*. Future studies using whole‐genome sequencing will be key to resolving remaining taxonomic uncertainties in this complex group.

## Author Contributions

**Xuan Pan:** conceptualization (equal), writing – original draft (equal), writing – review and editing (equal). **Xuming Wang:** investigation (equal), methodology (equal). **Robert W. Murphy:** writing – review and editing (equal). **Buqing Peng:** investigation (equal), methodology (equal). **Zongyun Zhang:** investigation (equal). **Rui Liao:** investigation (equal). **Shunde Chen:** conceptualization (equal), writing – review and editing (equal). **Shaoying Liu:** funding acquisition (equal), writing – review and editing (equal).

## Conflicts of Interest

The authors declare no conflicts of interest.

## Supporting information


**FIGURE S1:** ece371898‐sup‐0001‐Figures.docx.
**FIGURE S2:** ece371898‐sup‐0001‐Figures.docx.
**FIGURE S3:** ece371898‐sup‐0001‐Figures.docx.
**FIGURE S4:** ece371898‐sup‐0001‐Figures.docx.
**FIGURE S5:** ece371898‐sup‐0001‐Figures.docx.

**Table S1:** ece371898‐sup‐0002‐Tables.xlsx.

## Data Availability

The sequences used in this study have been deposited in the National Center for Biotechnology Information (NCBI) database. GenBank accession numbers are provided in the Table S2.
