# Supplementary material for: Two New Species of Ochotona (Mammalia, Lagomorpha) From Xizang, China
Source: Ecol Evol. 2025 Aug 6;15(8):e71898. doi: 10.1002/ece3.71898 (PMC12326426; doi:10.1002/ece3.71898)
Supplement: Supplementary file 1 — FIGURE S1: ece371898‐sup‐0001‐Figures.docx. FIGURE S2: ece371898‐sup‐0001‐Figures.docx. FIGURE S3: ece371898‐sup‐0001‐Figures.docx. FIGURE S4: ece371898‐sup‐0001‐Figures.docx. FIGURE S5: ece371898‐sup‐0001‐Figures.docx. [file ECE3-15-e71898-s002.docx]

**SUPPLEMENTARY MATERIAL**

**Two new species of *Ochotona* (Mammalia, Lagomorpha) from Xizang, China**

Xuan Pan ^1,2#^, Xuming Wang ^1#^, Robert W. Murphy ^3^, Buqing Peng ^1^, Zongyun Zhang ^4^, Rui Liao ^1^, Shunde Chen ^5*^, Shaoying Liu ^1*^

^1^Sichuan Academy of Forestry, Chengdu, Sichuan, China

^2^CAS Key Laboratory of Mountain Ecological Restoration and Bioresource Utilization & Ecological Restoration and Biodiversity Conservation Key Laboratory of Sichuan Province, Chengdu Institute of Biology, Chinese Academy of Sciences, Chengdu, Sichuan, China

^3^Department of Natural History, Royal Ontario Museum, 100 Queens Park, Toronto, ON, Canada

^4^Sichuan Forestry and Grassland Survey and Planning Institute, Chengdu, Sichuan, China

^5^Sichuan Normal University, Chengdu, Sichuan, China

^#^These authors contributed equally: Pan Xuan, Wang Xuming

^*^Correspondence: Chen Shunde([csd111@163.com](mailto:csd111@163.com)); Liu Shaoying ([shaoyliu@163.com](mailto:shaoyliu@163.com))

**SUMMARY**

**FIGURE S1** Phylogenetic trees based on 7 concatenated genes (mtDNA+nuDNA) and BI analyses. node supports are represented by Bayes values.

**FIGURE S2** Phylogenetic trees based on 5 concatenated genes (nuDNA) and BI analyses. node supports are represented by Bayes values.

**FIGURE S3** Phylogenetic trees based on 2 concatenated genes (mtDNA) and BI analyses. node supports are represented by Bayes values.

**FIGURE S4 P**hylogenetic tree based on CYTB dataset and ML and BI analyses; node supports are represented by ultrafast bootstrap/SH-aLRT/Bayes values.

**FIGURE S5** PCA plots of morphometric analysis based on 16 craniodental measurements.

**FIGURE S1** Phylogenetic trees based on 7 concatenated genes (mtDNA+nuDNA) and BI analyses. node supports are represented by Bayes values.

**FIGURE S2** Phylogenetic trees based on 5 concatenated genes (nuDNA) and BI analyses. node supports are represented by Bayes values.

**FIGURE S3** Phylogenetic trees based on 2 concatenated genes (mtDNA) and BI analyses. node supports are represented by Bayes values.

**FIGURE S4 P**hylogenetic tree based on CYTB dataset and ML and BI analyses; node supports are represented by ultrafast bootstrap/SH-aLRT/Bayes values.

**FIGURE S5** PCA plots of morphometric analysis based on 16 craniodental measurements.
